# Activation of p62/SQSTM1–Keap1–Nuclear Factor Erythroid 2-Related Factor 2 Pathway in Cancer

**DOI:** 10.3389/fonc.2018.00210

**Published:** 2018-06-07

**Authors:** Yoshinobu Ichimura, Masaaki Komatsu

**Affiliations:** Department of Biochemistry, Niigata University Graduate School of Medical and Dental Sciences, Niigata, Japan

**Keywords:** selective autophagy, p62/SQSTM1, Keap1–nuclear factor erythroid 2-related factor 2 system, metabolic reprogramming, cancer

## Abstract

Autophagy and the Keap1–Nrf2 system are major cellular defense mechanisms against metabolic and oxidative stress. These two systems are linked *via* phosphorylation of the ubiquitin binding autophagy receptor protein p62/SQSTM1 in the p62–Keap1–Nrf2 pathway. The p62–Keap1–Nrf2 pathway plays a protective role in normal cells; however, recent studies indicate that this pathway induces tumorigenesis of pre-malignant cells, and promotes the growth and drug resistance of tumor cells *via* metabolic reprogramming mediated by Nrf2 activation. These findings suggest that impairment of autophagy is involved in the acquisition of malignancy and maintenance of tumors, and furthermore, that p62/SQSTM1 could be a potential target for chemotherapy in cancers that harbor excess p62.

## Introduction

**Figure 1:**
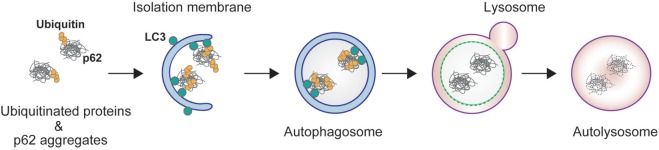
p62-mediated selective autophagy. p62 interacts with ubiquitinated proteins through its C-terminal ubiquitin-associated (UBA) domain, and forms aggregates through its N-terminal Phox and Bem1 domain. The resulting protein aggregates are tethered to the autophagosome by direct interaction of p62 and light chain 3 localized on the isolation membrane. The isolation membrane elongates and sequesters p62 and ubiquitin into the autophagosome. The autophagosome fuses with lysosome to form an autolysosome.

Autophagy is a bulk degradation process in which cytoplasmic components are sequestered in a double-membrane structure to form an autophagosome. The contents of the autophagosome are subsequently degraded after fusion with a lysosome (Figure 1) (1). Under starvation conditions, autophagy provides amino acids essential for protein synthesis in response to metabolic stress. Basal autophagy, on the other hand, removes specific substrates, including protein aggregates, damaged organelles, and invading bacteria. This selective pathway uses autophagic receptor proteins for efficient degradation. Autophagic receptors are typically categorized into two different types: ubiquitin binding proteins and organelle membrane proteins. Ubiquitin binding receptors include p62/SQSTM1, neighbor of BRCA1 gene (NBR1), optineurin, nuclear dot protein 52 kDa/calcium binding and coiled-coil domain2 (CALCOCO2), Tax1 binding protein 1, and toll-interacting protein; membrane binding receptors include BCL2/adenovirus E1B 19 kDa interacting protein 3-like (NIX/BNIP3L), BNIP3, FUN14 domain containing 1, and family with sequence similarity 134, member B (FAM134B) (2). These receptors recognize and sort substrates, and recruit core autophagic machinery to the target at existing autophagosome formation sites.

**Figure 2:**
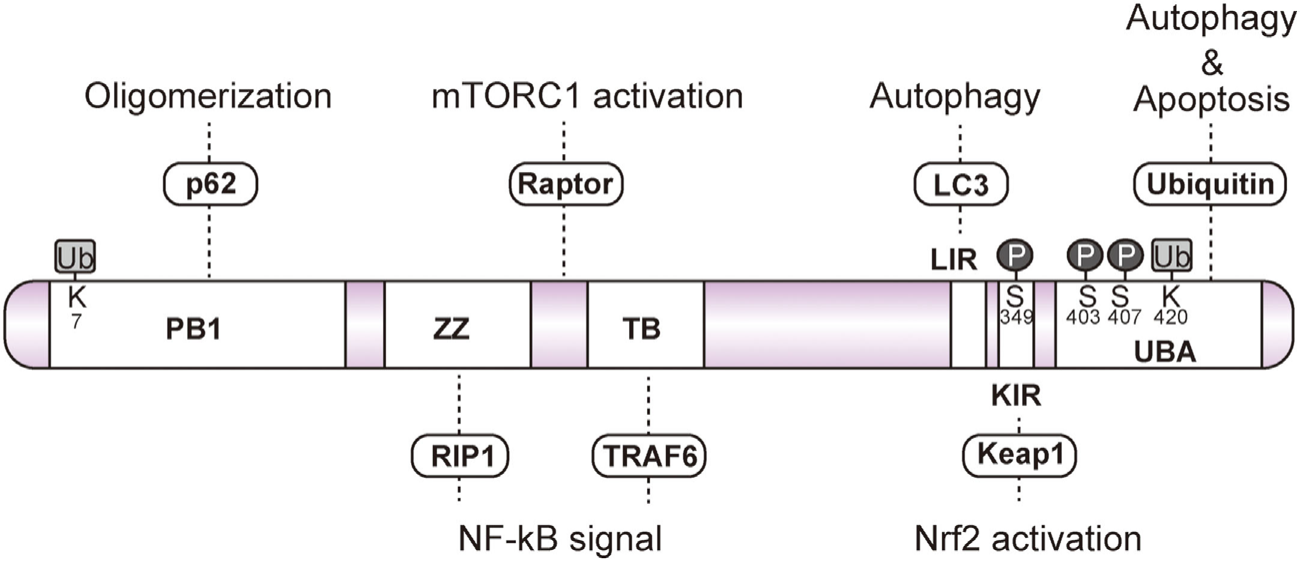
Schematic domain of p62. p62 forms a self-oligomer through its Phox and Bem1 (PB1) domain. The zinc finger (ZZ) and TRAF6-binding domains (TB) interact with RIP1 and TRAF6, respectively, to regulate NF-κB signaling. Raptor interacts with the unidentified region between ZZ and TB to activate mechanistic target of rapamycin complex 1. Light chain 3-interacting region and the C-terminal ubiquitin-associated domain (UBA) are essential for the sequestration of ubiquitinated substrates into the autophagosome. Phosphorylation of serine 403 and serine 407 residues in the UBA domain facilitates the interaction with ubiquitin. Phosphorylation of Keap1-interacting region at serine 349 enhances the interaction with Keap1. The UBA domain interacts with ubiquitinated caspase8 to induce apoptosis. Ubiquitination of the PB1 domain at lysine 7 abrogates p62 oligomerization. Ubiquitination of the UBA domain at lysine 420 disrupts the interaction with the ubiquitin chain.

Conserved among metazoans, the ubiquitin binding autophagy receptor p62/SQSTM1 (hereafter referred to as p62) acts as a hub protein in various cellular signaling pathways, including NF-κB, mechanistic target of rapamycin (mTOR), Caspase 8, and nuclear factor erythroid 2-related factor 2 (Nrf2) (3–7). p62 mediates the autophagic degradation of polyubiquitinated substrates *via* direct interaction with microtubule-associated protein light chain 3 (LC3) on the autophagosome (2) (Figure 1). p62 forms aggregates by self-oligomerization of its N-terminal Phox and Bem1 (PB1) domain, and associates with ubiquitin through its C-terminal ubiquitin-associated (UBA) domain (Figure 2). NBR1 has a remarkable similarity with p62 in the domain architecture, which consists of PB1, zinc finger, LC3-interacting region (LIR), and UBA domains (8). PB1 domain of p62 forms self-oligomer or hetero-oligomer with other PB1 proteins including NBR1, while PB1 domain of NBR1 forms hetero-oligomer only. Thus, NBR1 self-interacts through their coiled-coil domain, and cooperates with p62 oligomer in selective autophagy of ubiquitinated substrates (9). There are several examples of posttranslational modifications of p62 for selective autophagy. For example, phosphorylation of the p62 UBA domain at serine 407 by unc-51-like kinase 1 (ULK1) destabilizes the UBA dimer of p62, while sequential phosphorylation of serine 403 by casein kinase 2 (CK2), TANK-binding kinase 1 (TBK1), or ULK1 increases the affinity of UBA for ubiquitin chains (10–12). Ubiquitination of p62 at lysine 420 by the Keap1/Cul3 E3 ligase complex inhibits the dimerization of the UBA domain (13), while ubiquitination at lysine 7 in the PB1 domain by the E3 ligase TRIM21 suppresses the oligomerization of p62 (14).

Upon various cellular stress conditions, p62 functions as a signaling hub *via* characteristic domains, such as the zinc finger (ZZ) domain, TRAF6 binding (TB) domain, LIR, and Keap1-interacting region (KIR), which interacts with RIP kinase, TRAF6, Raptor, LC3, and Keap1, respectively (Figure 2). Transcription of p62 is activated by cellular stresses, such as oxidative, metabolic, and pathogenic conditions, and aberrant expression of p62 or defective autophagy causes appearance of large number of p62 aggregates into the cytoplasm (15, 16). Importantly, the aggregate of p62 has been found in a common hallmark in some of serious diseases, such as cancer, alcoholic hepatitis, and neurodegenerative disease (17). The aforementioned diseases are thought to result from loss or dysfunction of p62-regulated cell signaling.

## Pathophysiological Role of Autophagy in Cancer

Early research has indicated a role of autophagy in tumor suppression. Beclin 1, the mammalian ortholog of yeast Atg6, was reported as autophagy-related protein involved in tumorigenesis (18). Specifically, beclin 1 heterozygous-deficient mice were shown to exhibit increased frequency of spontaneous tumorigenesis in the liver, lung, and lymphomas (19, 20). Because systemic knockout of *Atg* genes in mice exhibit neonatal lethality due to deprivation of amino acids in plasma and tissue (21), conditional knockouts of *Atg* genes in mice have been used as a physiological model of autophagy. Intriguingly, mosaic knockouts of *Atg5* or liver-specific knockouts of *Atg7* in mice have demonstrated that autophagy deficiency generates benign tumors in the liver in an age-dependent manner (22, 23). Impairment of autophagy results in accumulation of damaged mitochondria, which are major sources of reactive oxygen species (ROS) resulting in DNA damage, thus contributing to malignant progression (24). Other studies report that reduction of autophagy results in growth suppression of various cancers, such as hepatocyte, pancreatic cancer, lung cancer, breast cancer, colon cancer driven by K-Ras or BRAF mutations (25). On the other hand, studies have found that overexpression of transcription factor EB, a critical regulator of autophagy, promotes cancer growth (26, 27). Autophagy-deficient tumors re-growth and form large tumors in allograft, suggesting that growth arrest of autophagy-deficient tumors is canceled through nutrient-generating autophagy in an ectopic environment (28). It has been reported recently that oxidation of p62 promotes its oligomerization *via* disulfide-linked conjugates, followed by activation of autophagy (29). In this pathway, p62 senses the ROS and induces autophagy for cellular homeostasis and cell survival even under the oxidative stress conditions in aging or cancer. Moreover, increased p62 in autophagy-defective cells inhibits RNF168, an E3 ligase for histone H2A activated in response to DNA damage (30). These findings suggest that autophagy-deficient cells abolish DNA repair activity thereby resulting in tumorigenesis. Autophagy plays a complex role in cancer, and its function can be dependent on the stage, environment, or type of cancer. Taken together, the above findings indicate that while autophagy plays a role in the inhibition of tumorigenesis, it could also facilitate the tumor growth once established, at least in mouse models.

## Keap1–Nrf2 Pathway

**Figure 3:**
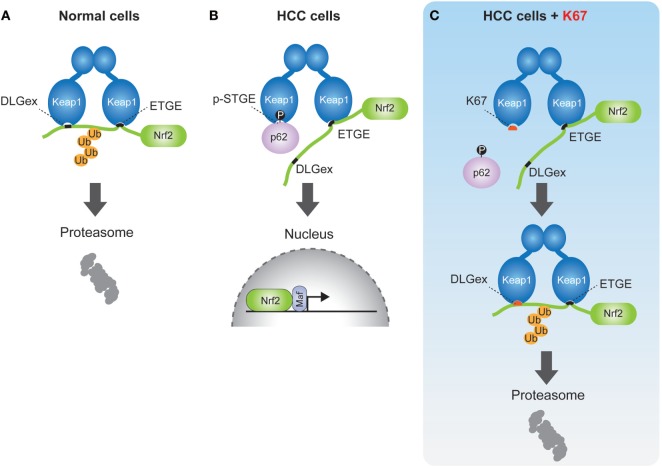
Schematic model of nuclear factor erythroid 2-related factor 2 (Nrf2) activation by phospho-p62 **(A)**. In normal cells, the ETGE and DLGex motifs of Nrf2 bind to the Keap1DC domain, which promotes ubiquitination of Nrf2 followed by its proteasomal degradation. **(B)** In human hepatocellular carcinoma cells, phospho-p62 (pS349) competitively interacts with the DLGex site on Keap1DC, resulting in the translocation of Nrf2 into nucleus. **(C)** K67 binds to Keap1DC at higher affinity than phosphor-p62, whereas the DLGex motif of Nrf2 binds to Keap1DC with covering K67. Nrf2 is eventually ubiquitinated and degraded by the proteasome.

Keap1–Nrf2 pathway is a critical cytoprotective response mediated by the activation of transcription factor Nrf2 during oxidative or electrophilic stress. Under normal conditions, Nrf2 is constitutively degraded in ubiquitin-proteasome system *via* the interaction with the E3 ubiquitin ligase adaptor protein Kelch-like erythroid cell-derived protein with CNC homology [ECH]-associated protein 1 (Keap1) (31). Keap1 binds to Nrf2 through direct interaction between the double glycine repeat or Kelch repeat (DGR) and the C-terminal region (CTR) of Keap1 (Keap1DC), and the ETGE and DLGex motifs of Nrf2. Known as the hinge and latch model (32–34), Keap1 forms a homodimer with its N-terminal BTB domain in which one protein interacts with high affinity to ETGE and the other with low affinity to DLGex (Figure 3A). Oxidative stress or electrophiles trigger a conformational change of Keap1 by modification of certain cysteine residues in Keap1, which leads to its dissociation from Nrf2 (35). The released Nrf2 translocates into the nucleus to induce the transcription of antioxidant-responsive element-regulated genes, such as the cytoprotective or metabolic-related genes *NQO1, HO-1, GCLC, GSTm*, which help to protect the cells from oxidative and metabolic stress.

## p62–Keap1–Nrf2 Pathway

In previous reports, we demonstrated that phosphorylation of p62 at serine 349 results in Nrf2 activation (6). p62 also has a KIR motif (349-STGE-352) (36), which allows binding to Keap1DC, but its affinity to Keap1 is significantly lower compared to the ETGE motif of Nrf2. During selective autophagy, p62 is translocated to ubiquitinated targets, such as protein aggregates, depolarized mitochondria, and pathogens, through the phosphorylation of the p62 UBA domain at serine 403 and serine 407 by CK2 and/or TBK1 (10, 11). Subsequently, mTOR complex 1 (mTORC1) phosphorylates p62 at serine 349, which dramatically enhances its interaction with Keap1, since the Keap1 binding affinity of phospho-p62 is higher than that of DLGex motif of Nrf2 (Figure 3B). Finally, the DLGex of Nrf2 is competitively displaced by phospho-p62, which results in the dissociation of Nrf2 from Keap1 and robust activation of Nrf2 (p62–Keap1–Nrf2 pathway) (Figure 3B) (6). p62–Keap1 and ubiquitinated cargos are eventually removed by selective autophagy. These results indicate that p62-mediated selective autophagy is coupled with the Keap1-Nrf2 system in normal cells. In p62–Keap1–Nrf2 pathway, the cytoprotective effects of Nrf2 activation could be enhanced in concert with the selective degradation of phosphorylated p62 and Keap1 complex.

## Dysregulation of the p62–Keap1–Nrf 2 Pathway in Cancer

Notably, persistent phosphorylation of mouse p62 at serine 351 (corresponding to serine 349 in human p62) has been found in hepatic adenoma in autophagy-deficient livers and in hepatitis C virus-positive human hepatocellular carcinoma (HCC) (22, 37). Knockout of *p62* in an HCC cell line markedly abrogates tumor growth, whereas forced expression of a phosphorylation-mimic allele of p62, but not a phosphorylation defective mutant, resulted in recovery of the growth defect (6, 22). These results indicate that the persistent activation of Nrf2 through phosphorylation of p62 is involved in the development of HCC. Importantly, Nrf2 also induces *p62* expression, resulting in the persistent activation of Nrf2 *via* a positive feedback loop in the p62–Nrf2–Keap1 pathway (16). Consistently, an amplified copy number of *p62* on chromosome 5q has been identified in renal cancer, suggesting that *p62* is an oncogene. Furthermore, accumulating evidence demonstrates that the abnormal expression of p62 is associated with malignancy in various cancers, including liver (22, 37), kidney (38, 39), lung (40), breast (41, 42), pancreatic (43), prostate (44, 45), head and neck (46, 47), ovarian (48, 49), oral (50, 51), colon (52, 53), endometrial (54), skin (55, 56), and gastric cancers (57). Indeed, accumulation of phosphorylated p62 has been observed in about half of HCC patients in our studies (6). Somatic mutations of Nrf2 and Keap1 have also been found in cancers at high frequency (58–60); these mutations could cause persistent activation of Nrf2 *via* disrupting the interaction between Nrf2 and Keap1. The above-mentioned lines of evidence suggest that dysregulation of p62–Keap1–Nrf2 pathway is involved in cancer development.

## Metabolic Reprogramming by the p62–Keap1–Nrf2 Pathway

**Figure 4:**
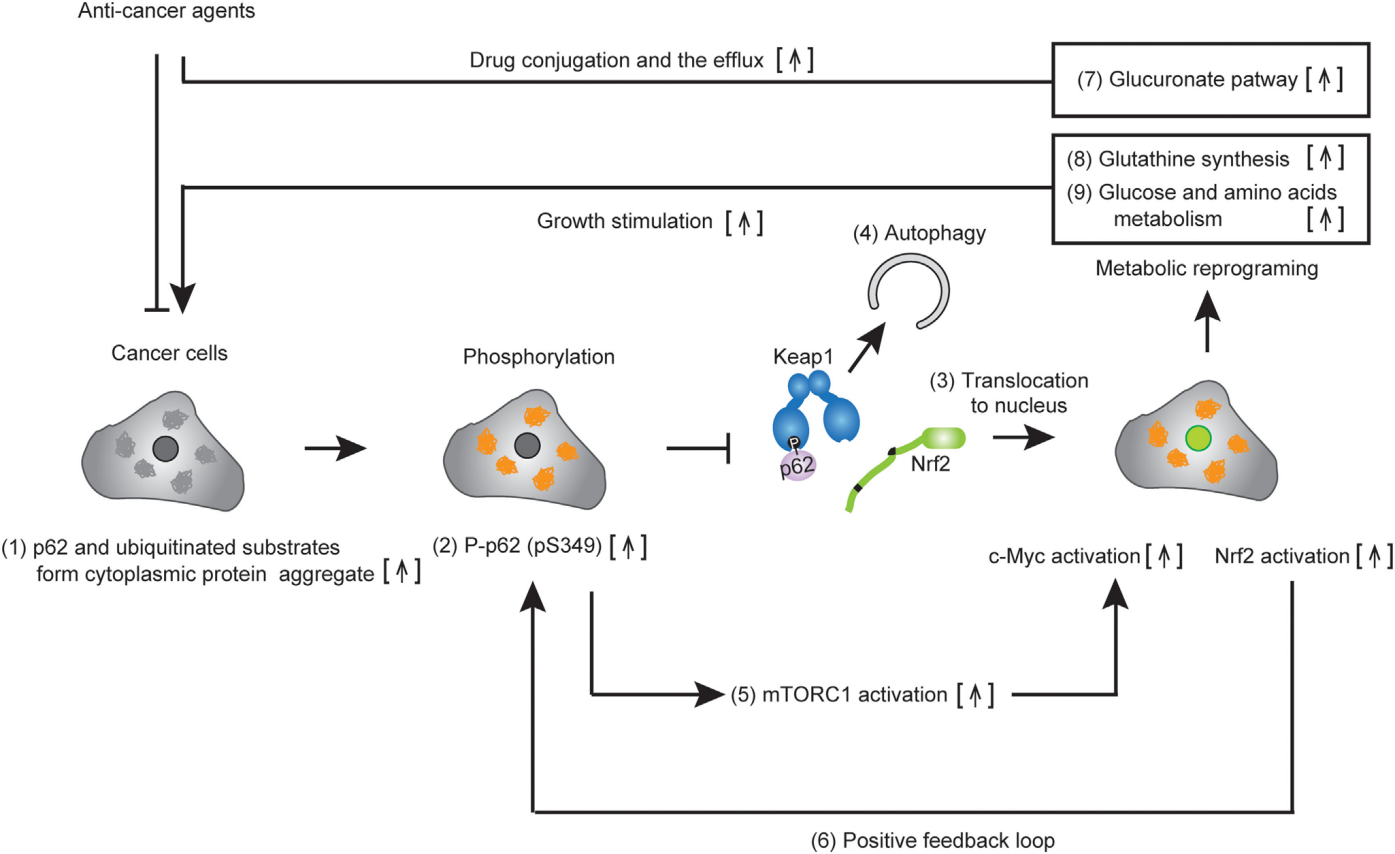
Metabolic reprogramming of cancer cells harboring phospho-p62. Accumulated p62 and ubiquitinated substrates form cytoplasmic protein aggregates in established tumor cells (1). The p62 localized in the protein aggregates are phosphorylated at serine 349 (2). Then, DLGex of nuclear factor erythroid 2-related factor 2 (Nrf2) is competitively displaced by phosphop-p62, which causes the dissociation of Nrf2 from Keap1. Nrf2 translocates to the nucleus and activates Nrf2-mediated gene expression (3), while the resulting p62–Keap1 complex is removed by p62-dependent selective autophagy (4). Increased p62 positively regulates c-Myc activity *via* the activation of mechanistic target of rapamycin complex 1 (5). Persistent activation of Nrf2 occurs due to the positive feedback loop of Nrf2 in cancer cells (6). Nrf2 activation induces metabolic reprogramming, including the glucuronate pathway (7) and glutathione synthesis (8), while c-Myc enhances glucose and glutamine metabolism (9). The activation of multiple metabolic pathways permits the drug resistance and cell proliferation of cancer.

Nuclear factor erythroid 2-related factor 2 has been shown to regulate the expression of antioxidant proteins, detoxification enzymes, proteasome subunits, and autophagy-related proteins for oxidative stress response and proteostasis (protein homeostasis) in normal cells. Recent studies demonstrate that Nrf2 increases the expression of multiple enzymes involved in the pentose phosphate pathway, purine nucleotide synthesis, as well as glutathione synthesis, and glutaminolysis in lung cancer, which activates the phosphatidylinositol 3-kinase-Akt pathway (61). In support of these findings, we also found that Nrf2 activation provided metabolic reprogramming of glucose and glutamine through the activation of Nrf2 in HCC harboring phosphorylated p62 (p-S349) (Figure 3B), which led to increased cell proliferation and resistance to anti-cancer agents of HCC (37) (Figure 4). Taken together, these findings suggest that molecular targeting of p62 represents a potential chemotherapeutic approach against HCC.

**Figure 5:**
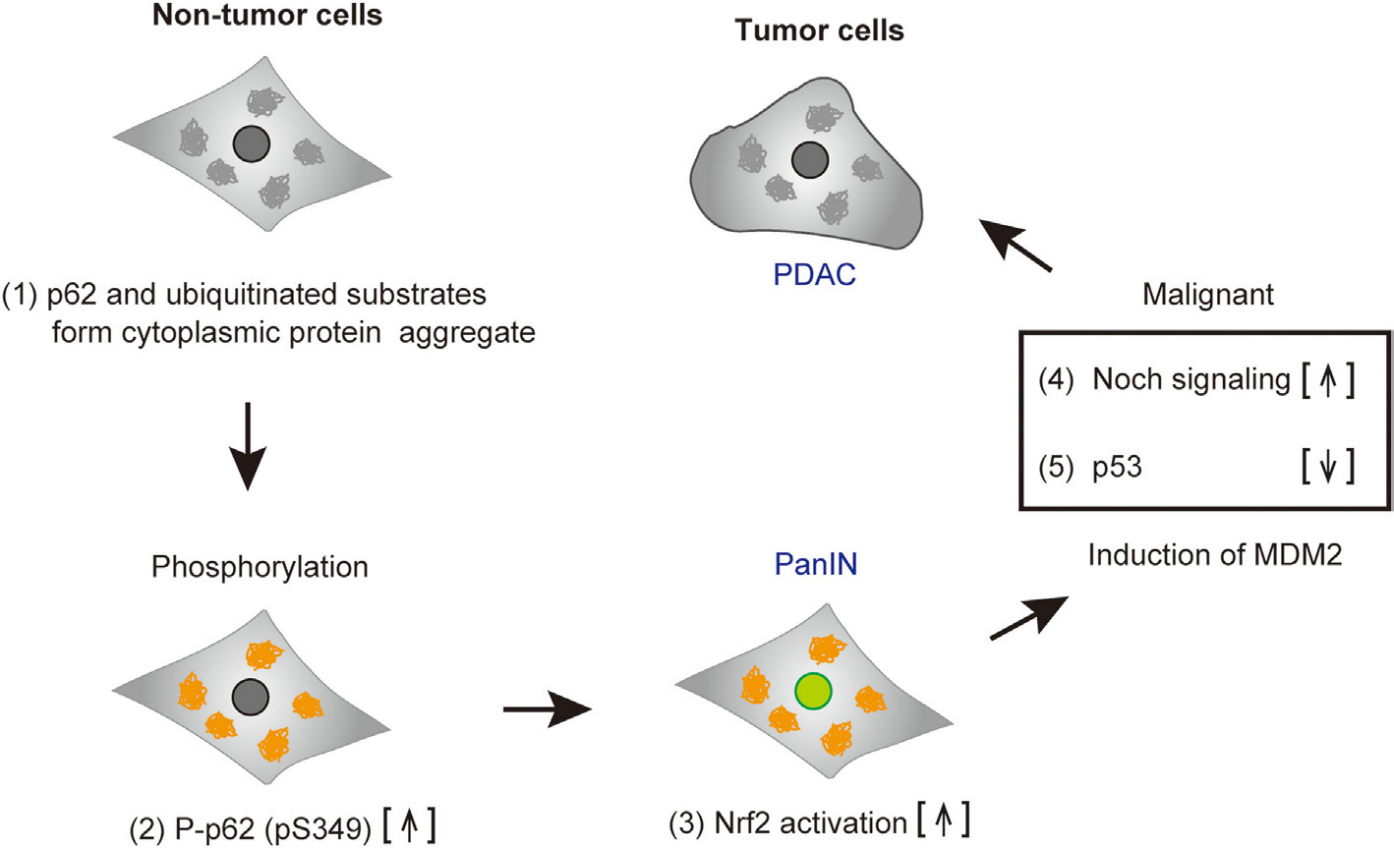
Tumorigenesis of cancer cells harboring phospho-p62. Accumulated p62 and ubiquitinated substrates form cytoplasmic protein aggregates in pre-malignant tumor cells (1). p62 is phosphorylated (2), and subsequently nuclear factor erythroid 2-related factor 2 (Nrf2) is activated (3). Mouse double minute 2 homolog (MDM2) induced by Nrf2 results in the increase of Notch signaling (4) and the reduction of p53, followed by cell malignancy (5). Abbreviations: PanIN, premalignant pancreatic intraepithelial neoplasia; PDAC, pancreatic ductal adenocarcinoma.

By chemical screening, we have identified an inhibitor for the Keap1-phosphorylated p62 (p-S349) protein–protein interaction—the acetonyl naphthalene derivative K67 (37) (Figure 3C). Structural analysis demonstrated that K67 binds to a Keap1DC pocket, which is the binding site of phosphorylated p62, Nrf2-ETGE, and Nrf2-DLGex. Treatment of HCC with K67 suppressed proliferation and reduced tolerance to cisplatin or sorafenib (37). Levels of p62 accumulation and c-Myc expression are reportedly associated with high risk for tumor recurrence and poor prognosis of HCC patients (62). Further, it has been demonstrated that high p62 expression in non-tumor tissue is required for transformation to HCC, which was caused by the activation of Nrf2, mTORC1, and c-Myc (62) (Figure 4). These results are consistent with the anti-proliferative and anti-malignant effects found in autophagy-deficient tumor cells (63). More recently, Karin and colleagues reported that p62-mediated activation of Nrf2 triggers mouse double minute 2 homolog (MDM2) expression in premalignant pancreatic intraepithelial neoplasia 1, resulting in development and malignancy of pancreatic ductal adenocarcinoma (43) (Figure 5).

## Conclusion and Future Perspectives

In recent years, there are many reports showing that p62–Keap1–Nrf2 pathway plays a protective role in oxidative and stress conditions. For example, ER stress-induced apoptosis is prevented by the activation of p62–Keap1–Nrf2 pathway (64). Quercetin attenuates the hepatotoxicity induced by a various hepatotoxicants, through the activation of p62–Keap1–Nrf2 pathway (65). Licochalcone A activates p62–Keap1–Nrf2 pathway and suppresses arthritis in a collagen-induced arthritis mouse model (66). Trehalose induces p62 expression and activates p62–Keap1–Nrf2-mediated antioxidant response during oxidative stress (67). Meanwhile, persistent activation of p62–Keap1–Nrf2 pathway has been shown to be involved in liver tumorigenesis in mice (6, 22). More recently, we demonstrated that overexpression of a p62 variant lacking KIR mitigates Nrf2 activation (68). These results suggest that phopsho-p62 could be a novel target for cancer therapy as described in Ref. (37). Many kinase inhibitors have been used in cancer therapy. However, the protein kinases responsible for the phosphorylation of p62 in tumor have not yet been identified. Further studies are required to identify the regulatory factors involved in the p62–Keap1–Nrf2 pathway.

## Author Contributions

All authors listed have made a substantial, direct and intellectual contribution to the work, and approved it for publication.

## Conflict of Interest Statement

The authors declare that the research was conducted in the absence of any commercial or financial relationships that could be construed as a potential conflict of interest.
